# Genome and transcriptome sequencing characterises the gene space of *Macadamia integrifolia* (Proteaceae)

**DOI:** 10.1186/s12864-016-3272-3

**Published:** 2016-11-17

**Authors:** Catherine J. Nock, Abdul Baten, Bronwyn J. Barkla, Agnelo Furtado, Robert J. Henry, Graham J. King

**Affiliations:** 1 Southern Cross Plant Science, Southern Cross University, Military Road, NSW Lismore, 2480 Australia; 2 Queensland Alliance for Agriculture and Food Innovation, The University of Queensland, St Lucia, Qld 4072 Australia

**Keywords:** Macadamia, Proteaceae, Rainforest, Gene space, Genome, Transcriptome, Crop

## Abstract

**Background:**

The large Gondwanan plant family Proteaceae is an early-diverging eudicot lineage renowned for its morphological, taxonomic and ecological diversity. Macadamia is the most economically important Proteaceae crop and represents an ancient rainforest-restricted lineage. The family is a focus for studies of adaptive radiation due to remarkable species diversification in Mediterranean-climate biodiversity hotspots, and numerous evolutionary transitions between biomes. Despite a long history of research, comparative analyses in the Proteaceae and macadamia breeding programs are restricted by a paucity of genetic information. To address this, we sequenced the genome and transcriptome of the widely grown *Macadamia integrifolia* cultivar 741.

**Results:**

Over 95 gigabases of DNA and RNA-seq sequence data were *de novo* assembled and annotated. The draft assembly has a total length of 518 Mb and spans approximately 79% of the estimated genome size. Following annotation, 35,337 protein-coding genes were predicted of which over 90% were expressed in at least one of the leaf, shoot or flower tissues examined. Gene family comparisons with five other eudicot species revealed 13,689 clusters containing macadamia genes and 1005 macadamia-specific clusters, and provides evidence for linage-specific expansion of gene families involved in pathogen recognition, plant defense and monoterpene synthesis. Cyanogenesis is an important defense strategy in the Proteaceae, and a detailed analysis of macadamia gene homologues potentially involved in cyanogenic glycoside biosynthesis revealed several highly expressed candidate genes.

**Conclusions:**

The gene space of macadamia provides a foundation for comparative genomics, gene discovery and the acceleration of molecular-assisted breeding. This study presents the first available genomic resources for the large basal eudicot family Proteaceae, access to most macadamia genes and opportunities to uncover the genetic basis of traits of importance for adaptation and crop improvement.

**Electronic supplementary material:**

The online version of this article (doi:10.1186/s12864-016-3272-3) contains supplementary material, which is available to authorized users.

## Background

Early-diverging lineages can provide important insight into genomic evolution [1, 2]. The Proteaceae is a large Gondwanan plant family belonging to the ‘basal’ eudicots, a paraphyletic group comprising several lineages that diverged prior to the origin and spectacular radiation of largest clade of flowering plants, the ‘core’ eudicots [3, 4]. Extensive morphological and ecological diversity in the Proteaceae make it a focus for studies of adaptive radiation and biome evolution (e.g. [5–8]). The long-held view of rainforest ancestry for the Proteaceae is challenged by recent fossil evidence for a great diversity and abundance of major lineages in open, fire-prone habitats in central Australia during the late Cretaceous [9]. Although species diversity is highest in regions with Mediterranean climates including biodiversity hotspots in Southwest Australia and South Africa, generic diversity is highest in rainforests [10, 11].

Macadamia is the most economically important Proteaceae crop. The industry is based on cultivars developed from the Australian subtropical trees *Macadamia integrifolia*, *M. tetraphylla* and hybrids [12, 13]. Commercially-grown cultivars are diploid (2n = 28), highly heterozygous and closely-related to their wild progenitors [14–16]. All four *Macadamia* species are rare and threatened, and the lowland rainforest ecosystems to which they contribute are listed as critically endangered [17, 18]. The subtropical rainforests of eastern Australia are centres of plant endemism, with high rainfall and low fire frequency that acted as stable refugia through Quaternary glaciation and interglacial periods [19]. This habitat is in contrast to the open, fire-prone habitats that support the majority of extant Proteaceae species.

Rainforests are biodiverse and tree survival depends on long-term defense strategies to respond to the biotic stresses imposed by a broad range of insect herbivores and pathogens [20]. Genome sequencing of the rainforest fruit tree *Theobroma cacao* revealed an expansion of plant resistance (R) genes, and in particular a group of LRR-RLK receptor protein kinase genes involved in pathogen recognition [21]. In comparison to other eudicots, including the model tree *Populus trichocarpa*, there was also evidence for expansion of flavonoid and monoterpene-related genes involved in plant defense, insect resistance and floral scent. While little is known of the defense arsenal of *Macadamia*, cyanogenic glycosides have been identified and cyanide has been detected in seedlings [22, 23]. Cyanogenesis is the production of hydrogen cyanide in response to wounding or attack by herbivores. Although this defense strategy is rare among plants including rainforest trees, it is more common in food plants and in the Proteacaeae subfamily Grevilleoideae to which *Macadamia* belongs [23–25]. Insect herbivores and fungal pathogens are a major cause of yield reduction in macadamia production and the identification of genes that may confer natural resistance would be of great benefit for crop improvement.

Whole genome sequences have been developed for many crop species accelerating the discovery of genes underlying agriculturally important traits [26, 27]. For perennial tree crops such as macadamia with long generation times, selective breeding is a protracted and expensive process. Genomic information can improve the efficiency and precision of plant breeding through marker-assisted selection [28]. Sequence data for macadamia is very limited and the composition of the Proteaceae genome is unknown. Given its position as a large early-diverging eudicot family, its role as a model for adaptive radiation, and the economic importance of macadamia we aimed to characterise the gene space of *Macadamia integrifolia* through genome and transcriptome sequencing, assembly and annotation.

## Results

### Genome sequencing and assembly

A draft assembly of the *Macadamia integrifolia* cultivar HAES 741 was constructed with 51.57 Gb of quality-filtered short-read Illumina sequence data (Table 1). Preliminary *de novo* assembly of paired-end reads was improved by scaffolding with mate pair reads producing 193,493 scaffolds with a total assembly size of 518 Mb. The largest scaffold was 643,490 bp and N50 scaffold size was 4745 bp. The genome was estimated at 652 Mb in length based on a k-mer size of 26mer [29] suggesting that the assembly comprises 79% of the genome (Additional file 1: Figure S1).

**Table 1:** *Macadamia integrifolia* genome and transcriptome sequencing, assembly and annotation statistics

Library Type	Reads post QC *millions*	Nucleotides post QC *gigabases*
Genome sequencing:		
Illumina GAIIx 480 bp Insert (2x150 bp PE)	101.7	30.51
Illumina GAIIx 700 bp Insert (2x150 bp PE)	48.6	14.58
Illumina HiSeq 8000 bp Insert (2x100 bp MP)	32.4	6.48
Total	182.7	51.57
Transcriptome sequencing:		
Illumina HiSeq Flower (2x100 bp PE)	82.1	16
Illumina HiSeq Shoot (2x100 bp PE)	70	13.7
Illumina HiSeq Leaf (2x100 bp PE)	76	14.9
Total	228.1	44.6
Genome assembly	Contigs	Scaffolds
Number	210,726	193,493
Minimum size (bp)	388	500
Maximum size (bp)	379,349	643,490
N50 (bp)	3522	4745
Total assembly length (Mb)	477	518
Transcriptome assembly	Statistics	
Number of transcripts	298,030	
Maximum transcripts length (bp)	17,814	
Minimum transcript length (bp)	224	
Mean transcript length (bp)	823	
Standard deviation (bp)	886	
Total length (bp)	245,373,045	
N50 (bp)	1339	
Genome annotation	Statistics	
Number of gene models	35,337	
Average gene length (bp)	2518	
Average coding sequence length (bp)	1090	
Gene models similar to *Arabidopsis thaliana* TAIR10^a^	74%	
Gene models similar to *Nelumbo nucifera* ^a^	79%	
Eukaryotic 458 CORE genes available^a^	96%	

^a^BLASTP 1e-05

**Fig. 1:**
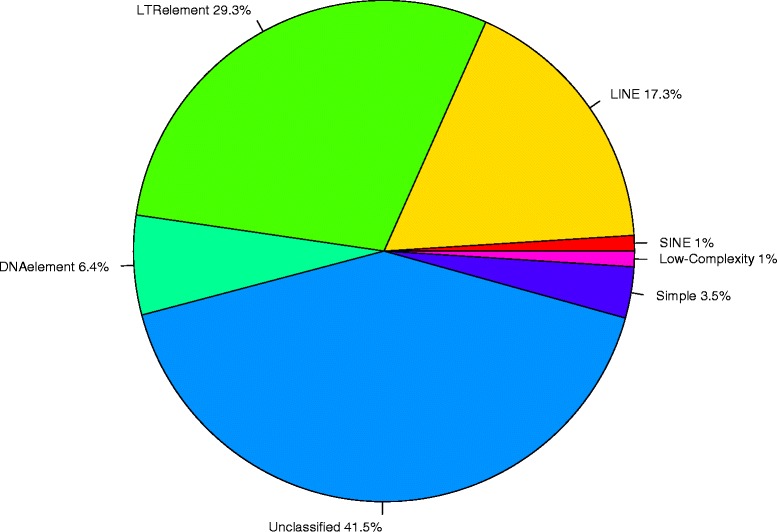
Repeat content of the macadamia genome showing the relative proportions of the long terminal (LTR), long and short interspersed (LINE, SINE), DNA element, simple, low-complexity and unclassified repeats identified using RepeatMasker and RepeatModeller

Approximately 37% of the assembled genome is identified as repetitive. As reported in most other plant species, long terminal repeats (Gypsy and Copia LTR) comprised the largest group accounting for approximately 29% of known repetitive elements and ~11% of the assembled genome (Fig. 1). Short and long interspersed repeats (SINEs and LINEs) accounted for ~18% while the majority of the identified repeats (~41.5%) were unclassified, lacking similarity to known repeats. In total, 98,114 perfect simple sequence repeat (SSR) motifs with di-, tri-, tetra-, penta- and hexanucleotide repeats were detected. Of these, 56,817 (57.9%) were dinucleotide repeats and consistent with reports for other plant species, the majority of these (58%) were AG/CT repeats [30]. In addition, there were 21,912 tri-, 11,262 tetra-, 5,045 penta- and 3,078 hexanucleotide repeats.

### Transcriptome assembly, gene prediction and functional annotation of proteins

Transcriptome assembly using the quality controlled reads from three cDNA libraries (flower bud, young leaf and shoot) in Trinity *de novo* generated 298,030 contigs (transcripts) including different isoforms per contig. The transcripts had an N50 size of 1339 bp, mean transcript length of 823 bp, maximum transcript length of 17,814 bp and minimum transcript length of 224 bp (Table 1). Initial transcripts were clustered to generate a final set of 244,925 transcripts, which were used as one source of evidence in the evidence-based gene model prediction pipeline. Final annotation using MAKER pipeline and assembled transcripts produced 35,337 high-confidence gene models. Of these, 90.3% (31,908) were supported by expression values, FPKM (fragments per kilobase of transcript per million mapped reads) of 1 or more, and 87.6% (30,940) were supported by at least two RNA-seq reads. Although 3430 gene models lacked RNA-seq read support it is important to note that RNA-seq data was collected from flower, leaf and shoot tissue only. Over 78 and 74% of predicted proteins had at least one significant BLASTP hit (1E-05) against *Nelumbo nucifera* or *Arabidopsis thaliana* proteins respectively.

Core eukaryotic genes (CEGs) are 248 highly conserved genes understood to be present in virtually all eukaryotes in a reduced number of paralogs [31]. Among flowering plants, 959 single copy genes have been identified that are shared between *Arabidopsis*, *Oryza*, *Populus* and *Vitis* [32]. More than 84% of these single copy genes (809 genes) and 96% of CEGs (237 genes) had a significant BLASTP hit (1E-05) against the predicted macadamia genes. Assessment of annotation completeness with BUSCO (benchmarking universal single-copy orthologs) [33] indicates that the macadamia gene space contains 77.4% of the expected content. Using a 429 single-copy eukaryote gene set, 192 complete single-copy, 90 complete duplicated, 140 fragmented, and 97 missing universal single-copy genes were identified. This compares to 94.6% (23 missing) and 89.7% (44 missing) of the expected content in the high-quality genome assemblies of *Eucalyptus grandis* and *Nelumbo nucifera* respectively. In total, 19,794 macadamia genes were assigned to 33,291 InterProScan (IPR) domains and 39,925 GO terms. Predicted macadamia genes with a significant BLAST hit in KASS (KEGG Automatic Annotation Server) were assigned to 349 known metabolic or signalling pathways. The metabolic pathway (ko01100) contained the largest number of genes (826), followed by biosynthesis of secondary metabolites (ko01110, 386 genes), biosynthesis of antibiotics (ko01130, 188 genes) and microbial metabolism in diverse environment (ko01120, 147 genes).

### Gene family analysis

**Fig. 2:**
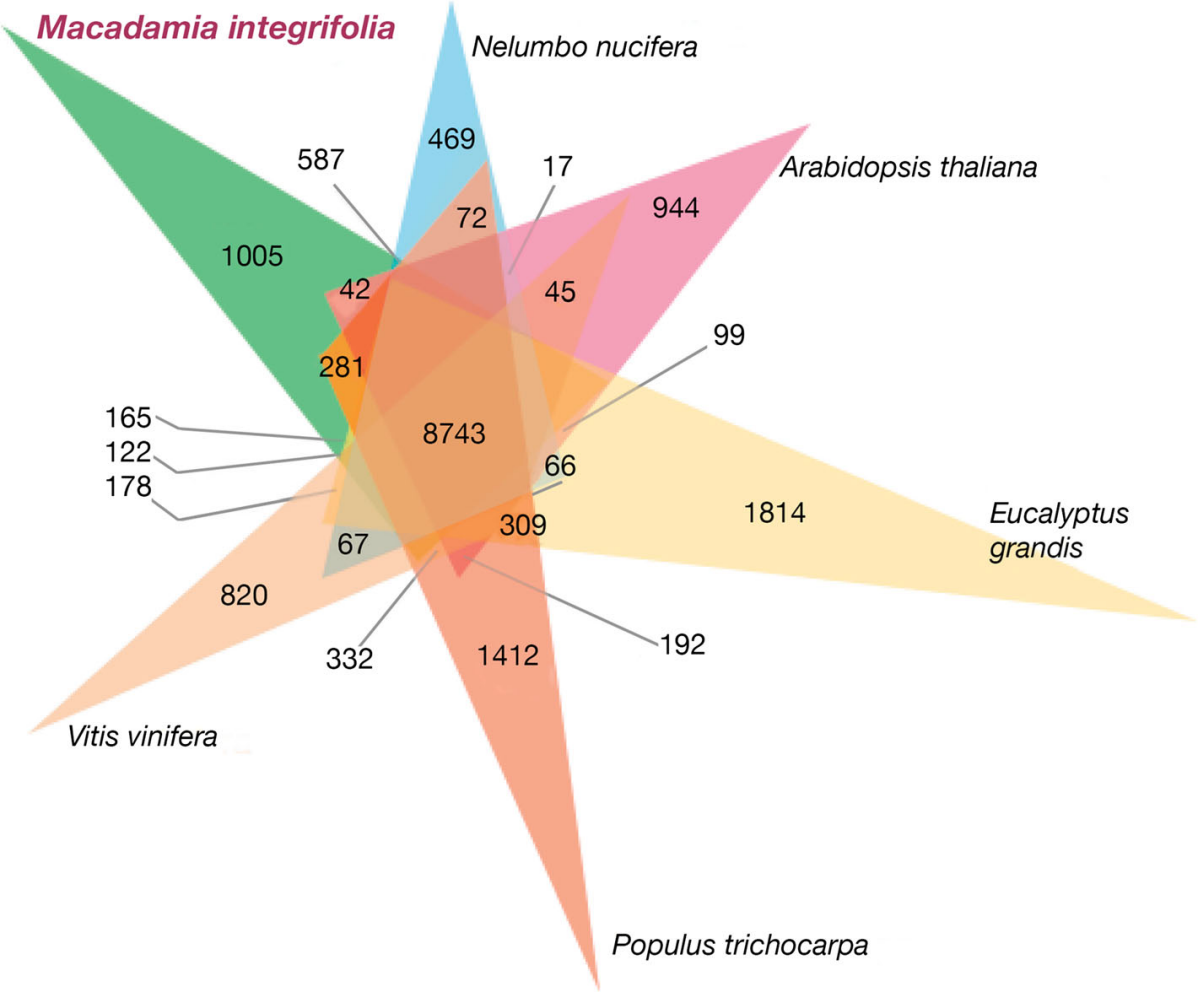
Venn diagram showing the distribution of gene families (orthologous clusters) among six plant species including early diverging eudicots *Macadamia integrifolia*, *Nelumbo nucifera* and core eudicots *Arabidopsis thaliana*, *Eucalyptus grandis*, *Populus trichocarpa* and *Vitis vinifera*

Comparative genome wide analysis of orthologous genes was performed with OrthoVenn [34] to compare putative *Macadamia integrifolia* protein sequences with those of five other eudicot species including the core eudicots *Arabidopsis thaliana, Eucalyptus grandis*, *Populus trichocarpa*, *Vitis vinifera* and the basal eudicot *Nelumbo nucifera*. In total, 207,057 sequences from the six species were grouped into 23,778 clusters. Of these, 17,314 clusters contained at least two species and 1412 were single copy clusters containing one gene for each of the six species. There were 8743 orthologous gene clusters shared across all six species indicating their conservation within eudicots, while 1005 clusters containing 3168 genes were specific to *Macadamia* (Fig. 2). *Macadamia* and *Nelumbo* shared 587 gene clusters, the highest between any two species compared, consistent with their positions among basal eudicot families.

Tests for GO enrichment of clusters unique to macadamia in comparison to other eudicot plant species identified 23 significantly enriched terms (*P* < 0.05), including ten related to biological processes (Table 2). Of these, the most significant terms (*P* ≤ 0.001) were the immune response-regulating signaling pathway (GO:0006898), detection of bacterium (GO:0016045), regulation of anion activity (GO: 0010359) and defense response signaling pathway (GO:0010204). One or more of these plant defense terms was assigned to 9 macadamia-specific clusters containing 28 predicted genes (Table 2). The best Swiss-Prot hits for these clusters were the LRR receptor-like serine threonine-protein kinases EFR and FLS2. In total, 22 and 11 macadamia gene models were functionally annotated as EFR and FLS2 genes respectively. Other GO terms associated with these clusters included plant-type hypersensitive response (GO:0009626), defense response to bacterium (GO:0006898) and defense response (GO:0006952). In total, 64 macadamia gene models with KEGG annotation were assigned to plant-pathogen interaction pathways for microbial defense through pathogen-triggered and effector-triggered immunity (Additional file 2: Figure S2).

**Table 2:** Hypergeometric test for significantly enriched biological process gene ontology (GO) terms of macadamia-specific gene clusters compared to those identified among six eudicot species

GO ID	Name	*p*-value	Macadamia specific		Six species, total	
			clusters	genes	clusters	genes
Plant Defense						
GO:0002764	immune response-regulatory signaling	1.06E-5	7	18	9	23
GO:0016045	detection of bacterium	3.61E-4	8	22	16	53
GO:0010359	regulation of anion channel activity	6.23E-4	8	24	17	59
GO:0010204	defense response signaling pathway	0.00102	9	28	18	86
Terpenoid synthesis						
GO:0016114	terpenoid biosynthetic process	0.03620	6	25	16	102
GO:0033383	geranyl diphosphate metabolic process	0.00367	3	10	3	10
GO:0043693	monoterpene biosynthetic process	0.01299	3	14	4	44
GO:0006200	obsolete ATP catabolic process	0.00653	4	9	5	10
GO:0009820	alkaloid metabolic process	0.02675	5	12	11	55
GO:0006075	(1- > 3)-beta-D-glucan biosynthetic process	0.03727	4	21	9	90

There was also evidence for an expansion of genes involved in terpenoid biosynthesis. In total, 78 macadamia gene models were functionally classified using Interpro as belonging to the terpene synthase gene family. Of these, 30 had high protein sequence similarity (1E-025) in BLASTP comparisons to *Arabidopsis thaliana* TPS-b monoterpene synthases. Among macadamia-specific clusters, significantly enriched GO terms included 25 predicted genes in six clusters involved in terpenoid biosynthetic process (GO:0016114, *P* = 0.036), and in particular biosynthesis of monoterpenes through geranyl diphosphate metabolic process (GO:0033383, *P* = 0.004) and monoterpene biosynthetic process (GO:0043693, *P* = 0.013). Monoterpenes, or C_10_ isoprenoids are components of essential oils and fragrance in aromatic plants with roles in pollinator attraction, plant-plant interaction and defense with potential as pesticides and antimicrobial agents. While the functionality of these putative genes is yet to be tested, these results suggest that there may have been a lineage-specific expansion in macadamia of gene families involved in monoterpene synthesis.

### Identification of candidate genes potentially involved in cyanogenic glycoside biosynthesis

**Fig. 3:**
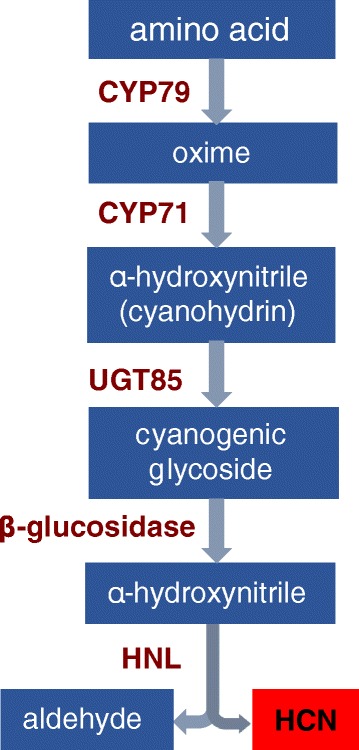
Overview of the biosynthetic and catabolic pathway of cyanogenic glycosides in plants

In *Macadamia*, the cyanogenic glycoside (CG) dhurrin and its diglucoside derivative proteacin have been identified [23]. The metabolic pathways for cyanogenesis are best understood in *Sorghum bicolor*, *Trifolium repens* and *Prunus spp.* with three genes (CYP79, CYP71 and UGT85) encoding enzymes in the CG biosynthesis pathway from amino acid. Synthesis from specific amino acids is catalysed by cytochrome P450s and UDP-glucosyltransferase. Cyanogenesis occurs upon tissue disruption with catabolism involving a β-glucosidase and release of hydrogen cyanide (HCN) that is either catalyzed by a α-hydroxynitrile lyase (HNL) or occurs spontaneously at high pH (Fig. 3).

We identified 11 macadamia gene models with high sequence similarity to those encoding CYP79, CYP71, UGT85, β-glucosidase and HNL in cyanogenic plants *Sorghum bicolor, Trifolium repens* and *Prunus spp.* and in the model plant *Arabidopsis thaliana* (Table 3). Mapping of global RNA-seq reads from young leaf, shoot and flower bud to macadamia candidate genes provided limited evidence of expression for some, and strong evidence for others with up to 53,071 reads per gene mapped at expression values (fragments per kilobase of transcript per million mapped read, FKMP) ranging from 4.63 to 872.93 (Table 3). Based on the relatively high FKMP values and RNA-seq coverage of Maca026950-RA (CYP79), Maca027151-RA, Maca024545-RA (CYP71), Maca010817-RA, Maca026370-RA (UGT85) and Maca000104-RA (β-glucosidase) these genes are probable candidates for cyanogenic glycoside biosynthesis and catabolism in macadamia (Additional file 3: Figure S3).

**Table 3:** Candidate genes for cyanogenesis in macadamia

Macadamia gene model	FPKM	Similar to	BLASTP *E*-value
CYP79			
Maca026950-RA	872.93	Phenylalanine N-monooxygenase^a^	4.2E-177
		Tyrosine N-monooxygenase^b^	4.1E-172
		CYP79D15, AC gene^c^	7.7E-171
CYP71			
Maca027151-RA	756.71	CYP71B16 Cytochrome P450^a^	2.9E-083
Maca024545-RA	48.04	CYP71B20 Cytochrome P450^a^	2.6E-126
Maca026817-RA	36.31	CYP71B34 Cytochrome P450^a^	5.6E-120
Maca030139-RA	14.52	CYP71A1 Cytochrome P450^d^	2.1E-100
UGT85			
Maca010817-RA	29.91	UGT85A2 UDP-glycosyltransferase^a^	6.0E-176
Maca026370-RA	16.19	UGT85A2 UDP-glycosyltransferase^a^	1.3E-179
Maca030319-RA	9.96	UGT85B1 Cyanohydrin glucosyltransferase^b^	5.9E-119
		UGT85A2 UDP-glycosyltransferase^a^	4.5E-177
β-glucosidase			
Maca000104-RA	248.96	BGLU9 Beta-glucosidase^a^	9.0E-144
Maca007594-RA	14.20	Cyanogenic beta-glucosidase, LI gene^c^	1.4E-111
HNL			
Maca017028-RA	4.63	(R)-mandelonitrile lyase-like^a^	6.5E-194
		(R)-mandelonitrile lyase, MDL1 gene^e^	4.6E-074

^a^ *Arabidopsis thaliana*; ^b^ *Sorghum bicolor*; ^c^ *Trifolium repens*; ^d^ *Persea americana*;; ^e^ *Prunus dulcis*

## Discussion

Illumina shotgun sequencing was used to develop a draft assembly of *M. integrifolia*, the first for the large basal eudicot family Proteaceae. A *de novo* assembly was constructed with 51.57 Gb of quality-filtered DNA sequence data. Transcriptome assembly from 44.6 Gb of RNA-Seq data from leaf, shoot and flower tissue generated 244,925 transcripts. These were used as reference ESTs, and with the proteins of *Nelumbo nucifera* and *Arabidopsis thaliana* provided sources of evidence in the gene model prediction pipeline [35]. Using MAKER, 35,337 protein-coding genes were predicted of which over 90% were expressed in at least one of the green tissues examined. Subsequent evaluation of these gene models showed significant similarity to 96% of core eukaryotic genes [31] and 84% of single copy genes shared by the angiosperm taxa *Arabidopsis*, *Oryza*, *Populus* and *Vitis* [32] indicating that our assembly covers much of the functional gene space of macadamia. In comparison to the eudicots *Arabidopsis*, *Vitis*, *Populus*, *Eucalyptus* and *Nelumbo*, 1005 gene families were specific to macadamia. The closest available complete genome sequence, that of the aquatic sacred lotus *Nelumbo nucifera* [2], is over 110 million years divergent based on fossil evidence and dated molecular phylogenies [5, 36]. *Macadamia* and *Nelumbo* shared 587 gene clusters, the highest between any two taxa compared here and consistent with their relatively close taxonomic positions of Proteaceae and Nelumbonaceae among basal eudicot families.

Quality assessment of the draft genome assembly as determined by technical measurements including the number of scaffolds (193,493) and N50 (4745) indicate that it is fragmented in comparison to completed plant genomes, and further work is required to develop a more contiguous genome with scaffolds anchored to chromosomes. However, quality assessment based on expectations of gene content using BUSCO sets [33] indicate that 77.4% of the expected gene content is represented in our assembly. This compares to 94.6 and 89.7% in the comprehensively assembled and annotated genomes of *Eucalyptus grandis* [37] and *Nelumbo nucifera* [2] respectively. Ongoing efforts to improve coverage and reduce fragmentation include deeper short read genome sequencing, incorporation of longer PacBio reads, transcriptome sequencing of additional tissues and the development of a high-density genetic linkage map. *Macadamia integrifolia* is a diploid species with a haploid number of 14 chromosomes [14]. There are no previously published estimates of genome size. In a recent extensive assessment of Proteaceae genome size from flow cytometry-based estimates, a 60-fold range was reported. Most Grevilleiodeae species, however, had relatively small genome sizes with 1C values from 0.64 to 2.87 pg (~625 to 2800 Mb) genome [38]. The kmer-based estimate of 652 Mb from this study is relatively small compared to closely-related species, and suggests that the draft assembly spans approximately 79% of the genome.

### Evidence for expansion of plant defense-related gene families

Rainforests are among the oldest and most diverse ecosystems [39]. Australian subtropical rainforests in particular, are ancient refugia with high levels of plant species richness, endemism and rainfall [19]. Recent evidence suggests that insects and pathogens are instrumental in the maintenance of plant species diversity in rainforests [40]. Likewise, elevated predator-pathogen pressure is hypothesised to increase and diversify plant chemical defense systems. Plants have developed a wide range of defense systems to respond to the biotic stresses exerted by the predators and pathogens with which they have co-evolved [41, 42]. Expansion of the receptor-like kinase genes in particular is purportedly in response to fast-evolving pathogens [43]. Comparative genomic analyses suggests that there has been a lineage specific expansion in macadamia of gene families with similarity to *Arabidopsis* LRR receptor-like serine threonine-protein kinases EFR and FLS2. These encode proteins that play a key role in pathogen recognition and the activation of plant defense response [44, 45] and it has been demonstrated that *Arabidopsis* EFR enhances bacterium resistance in dicot and monocot transgenic plants including rice [46]. Further research is needed to identify the complete suite of macadamia plant resistance and defense genes and to determine whether polymorphism at sites on candidate genes is associated with resistance to co-evolving pathogens in macadamia as has been previously reported in *Arabidopsis* [47, 48]. Future growth in macadamia global production is expected following rapid expansion of cultivation and demand particularly in Asia. Germplasm collections, including clones of wild and domesticated trees have been established. These resources, along with wild populations undoubtedly contain genetic variants of interest for breeding, including improved yield, nutritional benefits, pest resistance and capacity to grow under variable climatic conditions. Insect herbivores and microbial pathogens are a major cause of yield reduction in macadamia production and identification of natural resistance would be of benefit for crop improvement.

### Genes involved in cyanogenesis

Cyanogenesis is a plant chemical defense response to generalist herbivores involving the release of hydrogen cyanide following tissue disruption and hydrolysis of cyanogenic glycosides (CGs) [49, 50]. Endogenous recycling without cyanide release suggests that CGs serve additional biological roles including nitrogen and carbon supply at specific plant developmental stages [51] and there is evidence that intermediate compounds produced during biosynthesis of CGs have anti-microbial activity [52–54]. While relatively few plant species are cyanogenic, they are over-represented among food plants [25] and are common in the Proteacaeae, particularly in the subfamily Grevilleiodeae to which macadamia belongs [23, 24].

In macadamia, cyanide has been detected in seed, root, cotyledon and leaf tissue [22]. While levels in mature kernels of the commercial species *M. integrifolia* and *M. tetraphylla* are extremely low, they are much higher in the bitter mature kernels of *M. ternifolia* and *M. jansenii*. In almond *Prunus amygadalus*, bitterness of the kernel is determined by the content of the cyanogenic diglucoside amygdalin [55]. Intraspecific and temporal variation in cyanogenic capacity, and acyanogenic individuals have been reported in a number of cyanogenic plant taxa (e.g. [56, 57]). In white clover *Trifolium repens*, inheritance follows a Mendelian two-locus model. The *Ac/ac* (CYP79D) gene controls production of cyanogenic glycosides, and the *Li/li* (cyanogenic β-glucosidase) gene controls their hydrolysis [58]. There is an apparent selective advantage for acyanogenic individuals in colder climates, and polymorphism is maintained within populations through recurrent gene deletions over time [59].

We identified macadamia homologues with high sequence similarity to genes encoding enzymes involved in CG biosynthesis in other cyanogenic plants including *Sorghum bicolor* and *Trifolium repens.* Based on the relatively high RNA-seq expression values in green tissue, six homologues to genes encoding the enzymes CYP79, CYP71, UGT85 and β-glucosidase are probable candidates in macadamia to target for further analysis. The discovery of candidate cyanogenesis genes in macadamia is likely to be an important step in facilitating the utilization of the smaller tree species *M. ternifolia* and *M. jansenii* into breeding programs to reduce tree size while retaining kernel edibility. In previous studies, 28% of the Proteaceae species tested were cyanogenic. This compares to 4.5% of 401 species from 87 families in Australian rainforests, and 4% of *Eucalyptus* species [24, 60]. The high proportion of cyanogenic plants in Proteaceae indicates that cyanogenesis is an important defense strategy in this family. Further work is planned to validate candidate genes, screen wild macadamia germplasm for natural variants and investigate the interaction between pest resistance, climatic variation and cyanogenesis in macadamia and more broadly across the Proteaceae.

## Conclusions

This study presents the first available genomic resources for the large basal eudicot family Proteaceae and provides a platform for comparative genomics. As a recently domesticated subtropical tree crop with a long generation time, macadamia presents unique challenges for crop improvement. Macadamia breeding and the utilisation of wild germplasm resources is presently restricted by a paucity of genomic information. We have assembled genome and transcriptome sequence data and here introduce the gene space of *Macadamia integrifolia* as a resource to access to most macadamia genes. This presents opportunities to uncover genes and markers associated with variation in traits of importance for conservation, domestication and crop improvement.

## Methods

### Plant materials

Fresh plant tissue was collected from a *Macadamia integrifolia*, cultivar 741 ‘Mauka’ individual from the Macadamia Varietal Trial plantation M2 at Clunes, New South Wales, Australia and stored at -80 °C. A voucher specimen is deposited in the Southern Cross University herbarium [accession PHARM-13-0813]. Prior to DNA and RNA extraction, leaf tissue was frozen in liquid nitrogen and ground using a tissue lyser (MM200, Retsch, Haan, Germany).

### Genomic DNA isolation and sequencing

Total genomic DNA was extracted using a DNeasy Plant Maxi kit (Qiagen Inc., Valencia, USA) for all DNA sequencing with the exception of mate pair (MP) library sequencing where DNA was extracted using a CTAB-based method developed for next-generation sequencing [61]. DNA was quantified using a Qubit dsDNA BR assay (Life Technologies, Carlsbad, USA). Genomic DNA was sheared using a Covaris S220 focused-ultrasonication device (Covaris Inc., Woburn USA). Paired-end libraries (PE) with average insert sizes of 480 and 700 bp and an 8 kb MP library were prepared using Illumina TruSeq DNA Sample Preparation kit v2 following manufacturer’s instructions (Illumina, San Diego, USA). Fragment size distribution and concentration were determined using a DNA 1000 chip on a Bioanalyser 2100 instrument (Agilent Technologies, Santa Clara, USA). PE and MP libraries were sequenced with Illumina GA IIx (150 x 2 cycles) and HiSeq 2000 (100 x 2 cycles) instruments respectively.

### Genome assembly and scaffolding

Paired-end sequence reads were trimmed to remove low quality bases and adapter sequences and *de novo* assembled using CLC Genomics Workbench (CLC) version 6.5 (CLC Bio, Aarhus, Denmark) that has been used in the assembly of plant genomes including Norway spruce *Picea abies* [62] and barley *Hordeum vulgare* [63]. CLC *de novo* assembler, which utilizes de Bruijn graphs, was used for assembly of Illumina PE reads with the option to map reads back to contigs following previously described parameters [4]. MP reads were also trimmed to remove low quality bases and adapter sequences. We observed very high proportion (>90%) of duplicated MP reads, presumably PCR duplicates, which were filtered using CLC. Genome assembly was performed in the following two steps: preliminary contig assembly using PE reads in CLC, followed by assembly of sequence contigs and filtered high quality MP reads using the scaffolding program SSPACE to obtain a final set of scaffolds [64]. Genome size was estimated based on k-mer analysis and depth of sequencing [29].

### Repetitive sequence analysis

RepeatModeler and RepeatMasker programs were used to identify repeats [65]. Putative repetitive sequences were identified using the RepeatModeler program with default parameters. In parallel, known repetitive sequences were identified using the RepeatMasker program with the latest release of RepBase curated repeat libraries [66]. Searches for simple sequence repeats (SSRs) were conducted using SciRoko [67] software with default parameters and ‘MISA’ mode.

### RNA extraction and transcriptome sequencing

To enable assembly of the transcriptome of macadamia, three tissues (leaf, shoot and flower) of cultivar 741 ‘Mauka’ were selected for deep RNA sequencing (RNA-seq). Total RNA was isolated from frozen tissue using Ambion Plant RNA Isolation Aid prior to extraction using an Ambion RNAqueous Kit following manufacturer’s recommendations (ThermoFisher Scientific, Waltham, USA). Libraries were prepared with Illumina TruSeq Stranded mRNA Library Preparation Kit and PE sequenced with an Illumina HiSeq 2500 (100 x 2 cycles).

### Transcriptome assembly

Quality control of tissue specific transcriptomic reads involved removal of low quality sequences, adapter sequences and empty reads using BBMap tools (sourceforge.net/projects/bbmap/). Retained high quality clean reads were assembled using the Trinity *de novo* transcriptome assembly program (version 2.0.2) with default parameters [68]. The Trinity *de novo* assembly pipeline consists of three different modules Inchworm, Chrysalis and Butterfly. Inchworm assembles short reads into unique sequences of transcripts. Chrysalis clusters the Inchworm transcripts and constructs de Bruijn graphs for each cluster where each cluster represents the full transcriptional complexity for a given gene. Butterfly then processes the individual graphs in parallel, tracing the paths that reads and pairs of reads take within the graph, ultimately reporting full-length transcripts for alternatively spliced isoforms, and resolving transcripts that corresponds to paralogous genes. The initial transcripts were clustered using the CD-hit-est [69] to generate final set of transcripts, which were used as one source of evidence in the evidence-based gene model prediction pipeline.

### Gene prediction and annotation

Annotation of gene models was conducted using MAKER (version 2.31.8) which is an evidence-based gene model prediction pipeline [70]. MAKER combines the power of protein and Expressed Sequence Tag (EST) based homology with *ab initio* gene predictions to produce polished gene annotations. Trinity assembled transcripts were used as reference ESTs, and proteins of *Nelumbo nucifera* and *Arabidopsis thaliana* were used as reference proteins [35]. Macadamia scaffolds were first repeat masked using RepeatMasker [65]. To obtain the homology based genes MAKER aligned reference ESTs and proteins using Blastx [71] and exonerate [72] against the macadamia scaffolds. *Ab initio* gene predictions were made by Augustus [73] and SNAP [74] gene prediction programs. MAKER created the final gene set by combining the evidence based and *ab initio* predictions.

### Functional annotation of proteins

Predicted protein coding genes were functionally annotated based on protein signatures and orthology relationships. Similarity search was performed against release (03-2015) of UniProt Swiss-Prot proteins. Functional domains, gene ontology (GO) terms, GO accessions were searched against InterPro using InterProScan software [75]. Functional and gene ontology (GO) domains were assigned using InterProScan as described in [76] with default parameters. InterProScan integrates a collection of protein signature databases including BlastProDom, HMMPfam, HMMSmart, HMMTigr, ProfileScan, HAMAP, PatternScan, SuperFamily, TMHMM, HMMPanther, Gene3D and Phobius. To inform biological interpretation of macadamia gene function, KEGG (Kyoto Encyclopedia of Genes and Genomes) reference pathway database was used to map macadamia genes to defined pathways [77]. The KASS (KEGG Automatic Annotation Server) was used to assign genes to metabolic pathways using BLASTX with an *E*-value cutoff of 1E-05 [78]. Tests for annotation completeness were conducted using BUSCO [33] with the eukaryote 429 gene set and compared results to those of the *Eucalyptus grandis* and *Nelumbo nucifera* genomes.

### Comparative genomic analysis and gene family identification

Protein sets of five plant species including core eudicots *Arabidopsis thaliana, Eucalyptus grandis, Populus trichocarpa*, *Vitis vinifera* and basal eudicot *Nelumbo nucifera,* were downloaded from respective public repositories. Along with the predicted macadamia proteins they were uploaded into the OrthoVenn web server for identification and comparison of orthologous clusters [34]. To identify orthologous groups OrthoVenn employs the OrthoMCL Markov clustering algorithm, although unlike OrthoMCL it employs UBLAST for the all-against-all similarity search, which is ~350 times faster than conventional BLAST [79]. Following clustering, orthAgogue [80] is used for the identification of putative orthology and inparalogy relations. To deduce the putative function of each ortholog, the first protein sequence from each cluster is searched against the non-redundant protein database UniProt [80] using BLASTP. Pairwise sequence similarities were determined among protein sequences of all species with a BLASTP *E*-value cut-off of 1E-05 and an inflation value of 1.5 for MCL. To test the quality and completeness of the gene space assembly of macadamia we identified orthologous clusters from analyses in OrthoVenn with Swiss-Prot hits to proteins reportedly involved in CG biosynthesis and activation, and conducted BLASTP searches of the macadamia candidates. In addition, reciprocal searches of protein sequences for five enzymes (CYP79, CYP71, UGT85, β-glucosidase and HNL) involved in CG biosynthesis from known cyanogenic plants were conducted with a DeCypher Tera-BLASTP search against all macadamia gene models.

## Additional files

Additional file 1: Figure S1. Kmer coverage plot for optimized kmer of 26 used to estimate haploid genome size of 652 Mb (600–700 Mb). Coloured curves correspond to the complete statistical model including erroneous and genomic kmers (red), using a diploid model, heterozygous kmers with major peak at 14 (green) and homozygous kmers with subpeak at 7 (blue). (PDF 5 kb)
Additional file 2: Figure S2. KEGG Plant-Pathogen Interaction pathway with mapping of macadamia genes, in red. (PDF 150 kb)
Additional file 3: Figure S3. RNA-seq read mapping to candidate genes for cyanogenesis in macadamia, including those encoding the cytochrome P450s CYP79 and CYP71, glycosyltransferase UGT85 and β-glucosidase. (PDF 535 kb)

## Abbreviations

BLAST: Basic local alignment search tool
bp: Base pair
BUSCO: Benchmarking universal single-copy orthologs
cDNA: Complementary DNA
CEG: Core eukaryotic genes
CG: Cyanogenic glycoside
EFR: Bacterial elongation factor Tu receptor
EPBC: Environment protection and biodiversity conservation
EST: Expressed sequence tag.
FLS2: Bacterial flagellin-sensing 2
FPKM: Fragments per kilobase of transcript per million mapped reads
GO: Gene ontology
HCN: Hydrogen cyanide
KASS: KEGG Automatic annotation server
KEGG: Kyoto encyclopedia of genes and genomes
LINE: Interspersed repeat
LTR: Long terminal repeat
Mb: Megabase
RNA-seq: RNA sequencing
SINE: Short interspersed repeat
SSR: Simple sequence repeat
