# Supplementary figures and images for: Genome and transcriptome sequencing characterises the gene space of Macadamia integrifolia (Proteaceae)
Source: BMC Genomics. 2016 Nov 17;17:937. doi: 10.1186/s12864-016-3272-3 (PMC5114810; doi:10.1186/s12864-016-3272-3)

k= 26

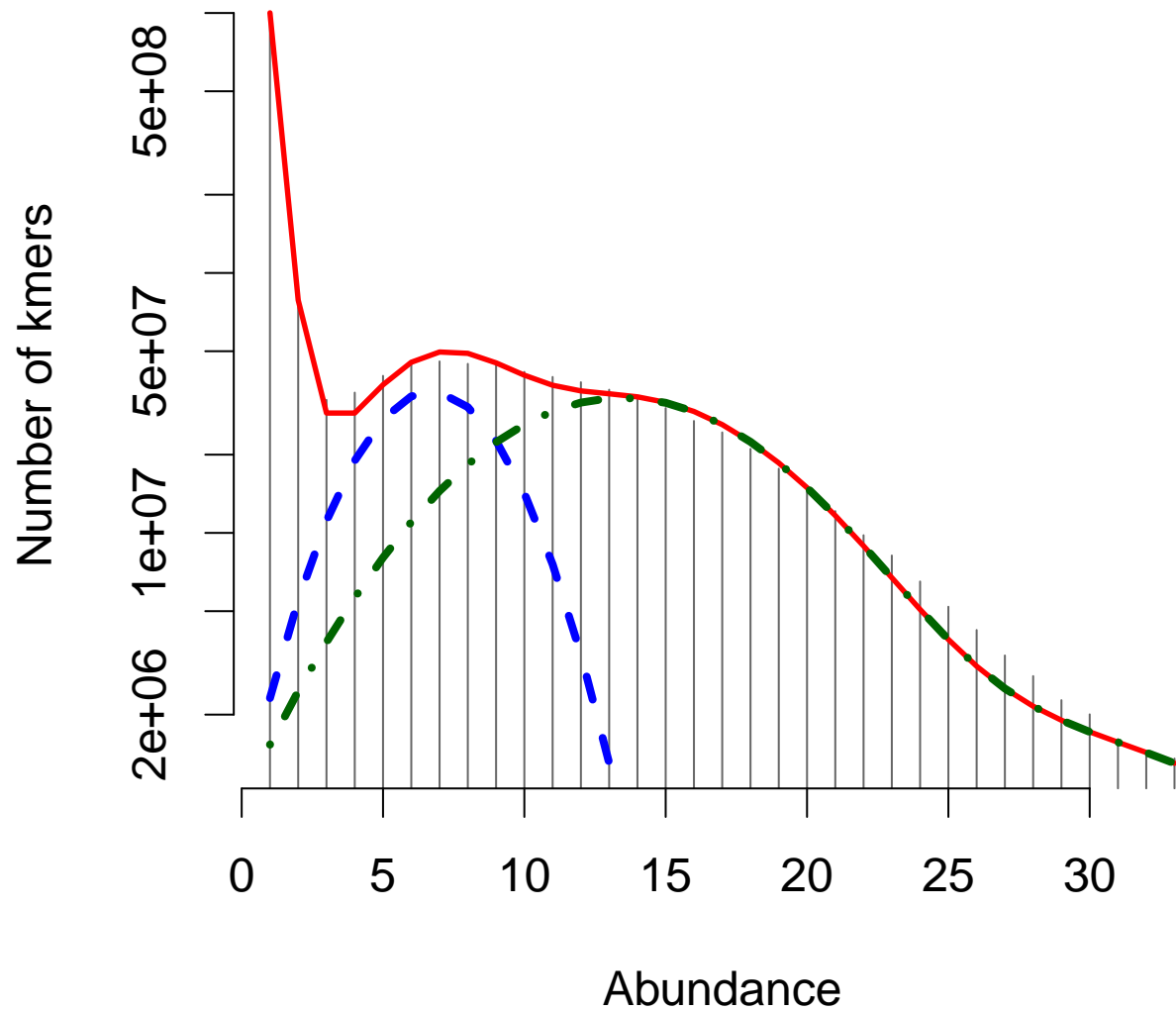

Supplement: Additional file 1: Figure S1. — Kmer coverage plot for optimized kmer of 26 used to estimate haploid genome size of 652 Mb (600–700 Mb). Coloured curves correspond to the complete statistical model including erroneous and genomic kmers (red), using a diploid model, heterozygous kmers with major peak at 14 (green) and homozygous kmers with subpeak at 7 (blue). (PDF 5 kb) [file 12864_2016_3272_MOESM1_ESM.pdf]

## PLANT-PATHOGEN INTERACTION

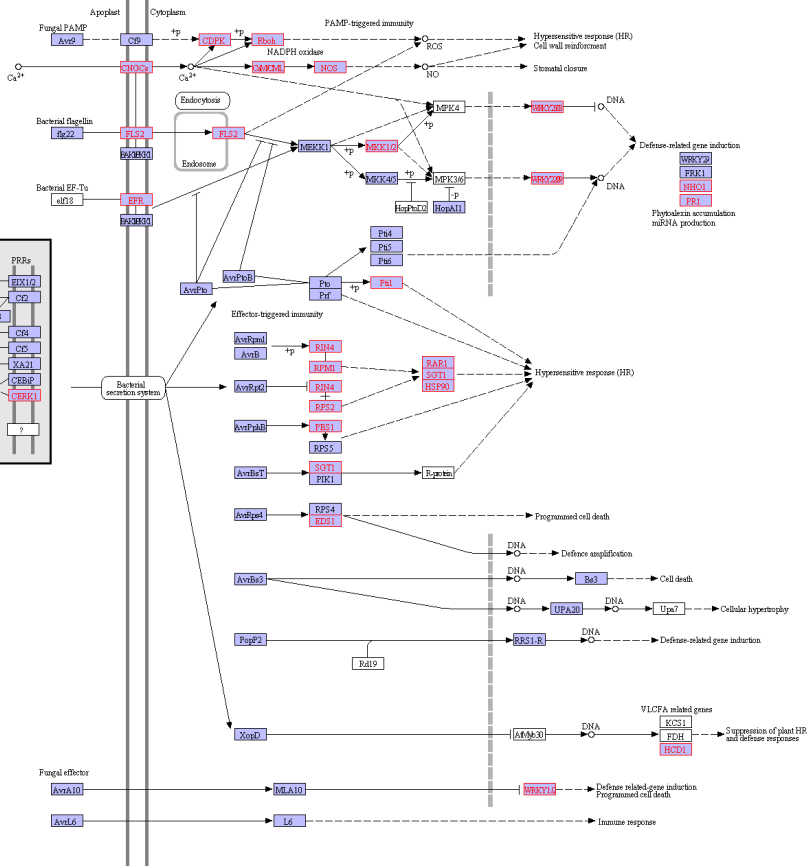

Supplement: Additional file 2: Figure S2. — KEGG Plant-Pathogen Interaction pathway with mapping of macadamia genes, in red. (PDF 150 kb) [file 12864_2016_3272_MOESM2_ESM.pdf]

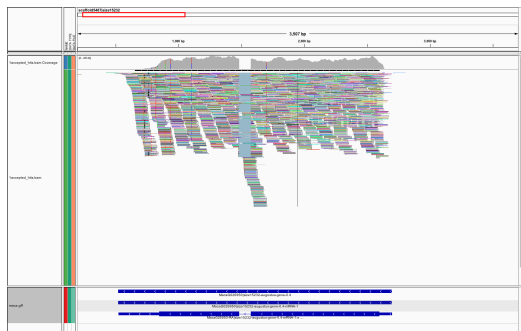

**CYP79** Maca026950-RA

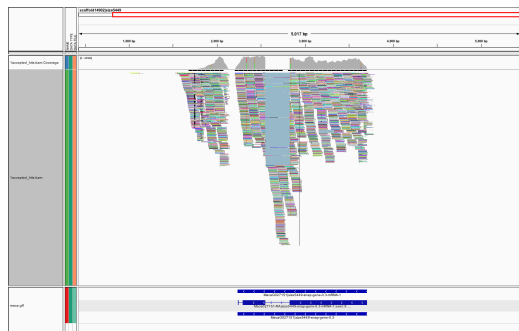

**CYP79** Maca027151-RA

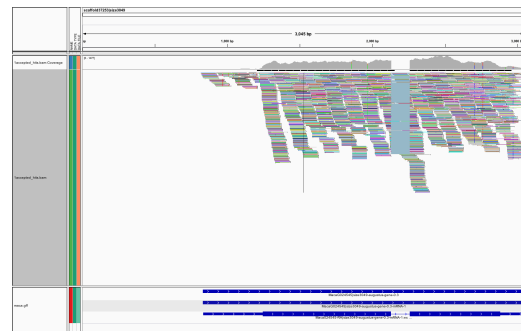

**CYP71** Maca024545-RA

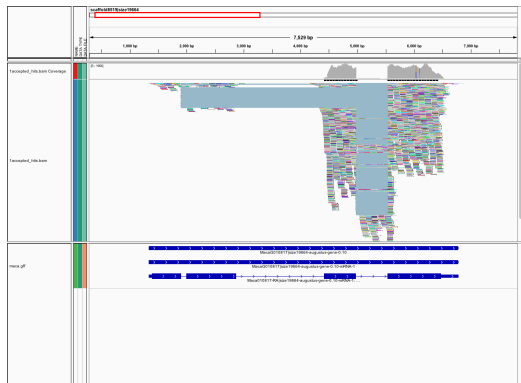

**UGT85** Maca010817-RA

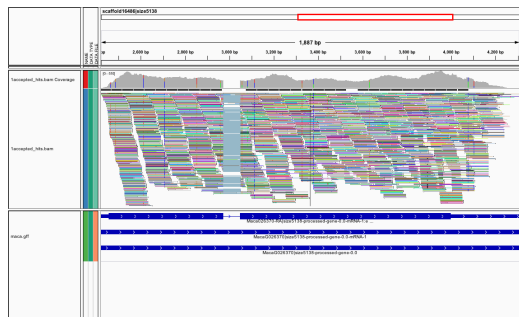

**UGT85** Maca026370-RA

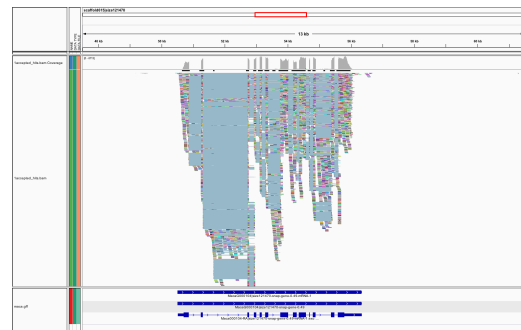

**$\beta$ -glucosidase** Maca000104-RA

Supplement: Additional file 3: Figure S3. — RNA-seq read mapping to candidate genes for cyanogenesis in macadamia, including those encoding the cytochrome P450s CYP79 and CYP71, glycosyltransferase UGT85 and β-glucosidase. (PDF 535 kb) [file 12864_2016_3272_MOESM3_ESM.pdf]
